# Ribosome-Inactivating and Related Proteins

**DOI:** 10.3390/toxins7051556

**Published:** 2015-05-08

**Authors:** Joachim Schrot, Alexander Weng, Matthias F. Melzig

**Affiliations:** Institute of Pharmacy, Freie Universitaet Berlin, Koenigin-Luise-Str. 2 + 4, 14195 Berlin, Germany; E-Mails: joachim.schrot@fu-berlin.de (J.S.); alexander.weng@fu-berlin.de (A.W.)

**Keywords:** ribosome-inactivating proteins, RIPs, type 1 RIP, RIP 1, type 2 RIP, RIP 2, *N*-glycosidase, nomenclature of RIPs, classification of RIPs

## Abstract

Ribosome-inactivating proteins (RIPs) are toxins that act as *N*-glycosidases (EC 3.2.2.22). They are mainly produced by plants and classified as type 1 RIPs and type 2 RIPs. There are also RIPs and RIP related proteins that cannot be grouped into the classical type 1 and type 2 RIPs because of their different sizes, structures or functions. In addition, there is still not a uniform nomenclature or classification existing for RIPs. In this review, we give the current status of all known plant RIPs and we make a suggestion about how to unify those RIPs and RIP related proteins that cannot be classified as type 1 or type 2 RIPs.

## 1. Introduction

Because of their *N*-glycosidase activity, ribosome-inactivating proteins inhibit protein synthesis by cleaving a specific adenine residue (A^4324^) from the 28S ribosomal RNA of the large 60S subunit of rat ribosomes followed by cell death [1]. In addition, certain RIPs can remove adenine from DNA and other polynucleotides for which reason they are also known as polynucleotide adenosine glycosidases [2]. PAP, an RIP from *Phytolacca americana*, can cleave not only adenine, but also guanine from the rRNA of *Escherichia coli* [3].

There are mainly two different types of RIPs: type 1 RIPs (RIP 1) and type 2 RIPs (RIP 2). Type 1 RIPs are single chain proteins, whereas type 2 RIPs consist of two polypeptide chains (A- and B-chain) that are usually linked through a disulfide bridge. The A-chain contains the enzymatic function and the B-chain has lectin properties enabling these proteins to bind to galactose residues on the cell surface. This facilitates the A-chain to enter the cell. Beside these different types of RIPs, there was the proposal to categorize an additional group of RIPs as type 3 RIPs including a protein from maize (b-32) and from barley (JIP60). The protein from maize, b-32, is synthesized as an inactive proenzyme, which is activated after the removal of an internal peptide segment obtaining two segments of 16.5 kDa and 8.5 kDa [4] that seem to act together as *N*-glycosidase. JIP60 consists of an amino-terminal domain resembling type 1 RIPs linked to a carboxyl-terminal domain, which has a similarity to eukaryotic translation initiation factor 4E [5,6]. Due to their different structures, these two proteins cannot be grouped into the classical type 1 RIPs. However, the necessity of denominating a new group of RIPs for only these two proteins is not realistic. Therefore, the suggestion was made to consider these two proteins as peculiar type 1 RIPs [7,8]. Beside the *N*-glycosidases, there is a second kind of RIPs belonging to the RNA hydrolase [9,10]. Both kinds of RIPs strongly inhibit the protein synthesis but show different mechanisms of action. The RNA hydrolases, like α-sarcin as the best-known representative, catalytically cleave a phosphodiester bond between G^4325^ and A^4326^ of the rat 28S rRNA. With the exception of crotin II, another representative of the RNA hydrolases (see Section 3.5), these kinds of RIPs are not described in detail in this review.

RIPs have mostly been found in plants, but the hypothesis that RIPs are ubiquitous should be discarded, because a gene encoding for an RIP has not been detected in the genome of *Arabidopsis thaliana* [11]. On the other hand, there are plants in which several RIPs occur simultaneously, and recently, it was shown that there are 31 genes in the rice genome encoding for type 1 RIPs [12]. Beside the plant RIPs, a type 1 RIP was also found from the species algae *Saccharina japonica*, which were denominated as lamjapin [13]. In addition, researchers are also aware of some type 1 RIPs from fungi, such as pleuturegin from *Pleurotus tuberregium* [14], lyophyllin from *Lyophyllum shimeji* [15], flammutin and velutin from *Flammulina velutipes* [16], hypsin and marmorin from *Hypsizygus marmoreus* [17,18], and volvarin from *Volvariella volvaceae* [19]. There are also two type 1 RIPs from bacteria: shiga toxin from *Shigella dysenteria* [20], and verotoxin 1 (shiga-like toxin) from *Escherichia coli* [21]. At last, adenine glycosylase activity was even found in some mammalian tissues [2].

RIPs show several enzymatic activities, such as chitinase activity [22], superoxide dismutase activity [23], DNase activity [24], and lipase activity [25]. Due to the *N*-glycosidase activity on viral RNA, RIPs have an antiviral effect, which is considered as a physiological function. But the enzymatic activity could also be related to a role in the defense of plants against predators and fungi [7,8,26]. Because of the *N*-glycosidase activity on genomic plant DNA, it is also believed that RIPs could play an undefined role in plant senescence [27]. RIPs might also give the plants evolutionary advantages as a kind of protection under unfavorable situations [28]. Anyway, no precise biological role has yet been assigned to RIPs [29], but most of the authors favor the antiviral role. Thus, in agriculture, research was performed to increase the resistance against viruses by using DNA recombinant technology (reviewed in [11]). In medicine, research for treatment of HIV diseases was performed leading to phase II study [30]. But most research of the use of RIPs is aimed at anti-cancer therapy in leading RIPs selectively to malignant tumor cells to be eliminated. Therefore, type 1 RIPs and the A-chains of type 2 RIPs are coupled to antibodies or other targeting moieties like growth factors, other hormones or smaller peptides generating targeted toxins [31,32,33]. These conjugates, however, contain highly potent toxins with a high potential of side effects, because they are partly taken up non-specifically by macrophages or other somatic cells. Another issue regarding the application of these conjugates in an anti-cancer therapy is the response of the immune system, because they are antigens. To reduce at least the high potential of side effects, it is necessary to begin the dosage of these conjugates as low as possible. That seemed to be possible since a synergistic effect of saponins and type 1 RIPs increasing the toxic effect of type 1 RIPs drastically [34,35,36] has been discovered. For that, the saponins must consist of certain molecule units [37], and it has been found that the synergistic effect is not based on stimulating phagocytosis [38], but increasing the endosomal escape in a certain way [39,40]; thus, the type 1 RIPs enter the cytosol.

In the last decade, several reviews about RIPs were published setting the focus on the chemical and biological properties and activities, distribution in nature or possible use of the RIPs (e.g., [8,11,41,42,43,44]). There is one review that contains a table of all hitherto known RIPs [7]. During our investigations, we found that this table needs to be added with several more RIPs and RIP related proteins. Moreover, we found that some proteins were designated with different terms, e.g., nigrin b from *Sambucus nigra* or sieboldin-b from *Sambucus sieboldiana* were also designated as SNA-V or SSA-b-2, respectively. In addition, in some cases, the same term was used to designate different proteins, e.g., the term momordin II was used for a protein from *Momordica balsamina* as well as for a protein from *Momordica charantia* or the term MAP was used for a protein (MAP 30) from *Momorica charantia* and for a protein from *Mirabilis jalapa* (MAP = *Mirabilis* antiviral protein). These examples are intended to illustrate that there is still no unambiguous nomenclature for the RIPs. There are also ambiguities about the classification of some proteins, whether they are type 2 RIPs or just lectins, because no assay concerning the toxicity was performed or there was no information given about the structure: SGSL from *Trichosanthes anguina*, TCSL from *Trichosanthes cucumerina*, TKL-1 from *Trichosanthes kirilowii*, TDSL from *Tichosanthes dioica*, and BDA from *Bryonia dioica*. At least since the knowledge that RIPs and lectins evolved from common ancestral genes [29], it is very likely that there are a number of other RIPs not detected to date. This assumption is corroborated by the investigation of several *Adenia* species, in which some new lectins were found, some of which may be referred to as type 2 RIP [45]. Therefore, with this review we created a summary table (Table 1) with all known RIPs and those proteins, which probably can be classified as RIPs, and we listed all terms that were used for the designation of these proteins. Since there is a phylogenetic relationship between RIPs and lectins, as mentioned above, we also listed the lectins from those plants, which are members of families that are known to include plants that synthesize one or more RIPs. For this, we focused on RIPs from plants, whereas other RIPs from algae, bacteria, and fungi are not considered further.

## 2. Table of RIPs from plants

**Table 1:** Summary table of ribosome-inactivating proteins (RIPs) and RIP related proteins from plants.

Family	Species ^1^	Protein	Classific.	Mw ^2^	IC_50_^3^	Source	References
**Adoxaceae**	*Sambucus ebulus* L.	Ebulitin α	RIP 1	32 kDa	10 ng/mL	leaves	[46]
		Ebulitin β	RIP 1	29 kDa	10 ng/mL	leaves	[46]
		Ebulitin γ	RIP 1	29 kDa	10 ng/mL	leaves	[46]
		Ebulin f	RIP 2	56 kDa	96 ng/mL; 0.3 nM (A) ^5^	green fruits	[29,47]
		Ebulin l	RIP 2	56 kDa	8.5 ng/mL; 0.15 nM (A) ^5^	leaves	[29,48,49]
		Ebulin r1	RIP 2	56 kDa	2.3 ng/mL	rhizomes	[49]
		Ebulin r2	RIP 2	56 kDa	2.3 ng/mL	rhizomes	[49]
		SEA	RIP 2	135,630 Da	1 nM	bark	[50]
		SEAII	lectin	33.5 kDa	-	rhizomes	[49]
		SELfd	lectin	68 kDa	820 ng/mL	green fruits	[47]
		SELld	lectin	67,906 Da	-	leaves	[51,52]
		SELlm	lectin	34,239 Da	-	young shoots	[53]
	*Sambucus nigra* L.	α-Nigritin	RIP 1	29 kDa	2.44–34 ng/mL	leaves	[54]
		β-Nigritin	RIP 1	40 kDa	2.44–34 ng/mL	leaves	[54]
		γ-Nigritin	RIP 1	27.5 kDa	2.44–34 ng/mL	leaves	[54]
		Nigritin f1	RIP 1	24,095 Da	100 ng/mL	green and mature fruits	[55]
		Nigritin f2	RIP 1	23,565 Da	100 ng/mL	mature fruits	[55]
		basic Nigrin b	RIP 2	63,469 Da	18 pg/mL; 0.3 pM (A) ^5^	bark	[56]
		Nigrin b = SNA-V	RIP 2	120 kDa	261 pM; 0.03 nM (A) ^5^	bark	[29,57,58,59]
		Nigrin f = SNA-Vf	RIP 2	120 kDa	1.9 ng/mL; 1.8 ng/mL; 0.03 nM (A) ^5^	fruits	[29,60,61,62]
		Nigrin l1	RIP 2	n.a. ^4^	n.a. ^4^	leaves	[63]
		Nigrin l2	RIP 2	n.a. ^4^	n.a. ^4^	leaves	[63]
		Nigrin s	RIP 2	57 kDa	~1 µg/mL	seeds	[64]
		SNA-I	RIP 2	240 kDa	150 ng/mL; 600 pM	bark	[58,65,66,67,68]
		SNA-I’	RIP 2	120 kDa	150 ng/mL	bark	[67,69]
		SNA-If	RIP 2	240 kDa	n.a. ^4^	fruits	[69,70]
		SNAflu-I	RIP 2	subunits of 30–33 kDa	n.a. ^4^	inflorescen-ces	[71,72]
**Adoxaceae**	*Sambucus nigra* L.	SNLRP1	RIP 2	62 kDa	0.5 µg/mL; 5.74 nM (A) ^5^	bark	[29,73,74]
		SNLRP2	RIP 2	60–62 kDa	n.a. ^4^	bark	[74]
		SNA-ld	lectin	n.a. ^4^	-	leaves	[63]
		SNA-lm	lectin	n.a. ^4^	-	leaves	[63]
		SNA-II	lectin	60 kDa	-	bark	[58,68,75]
		SNA-III	lectin	50 kDa	-	seeds	[58,76]
		SNA-IV = SNA-IVf	lectin	60 kDa	-	fruits	[58,62,77,78]
		SNA-IVl	lectin	n.a. ^4^	-	leaves	[63]
		SNApol-I	lectin	subunits of 26 kDa	-	pollen	[71]
		SNApol-II	lectin	subunits of 20 kDa	-	pollen	[71]
		TrSNA-I	lectin	22 kDa	-	bark	[70]
		TrSNA-If	lectin	22 kDa	-	fruits	[70]
	*Sambucus racemosa* L.	basic racemosin b	RIP 2	n.a. ^4^	n.a. ^4^	bark	[72]
		SRA	RIP 2	120 kDa	n.a. ^4^	bark	[72,79]
		SRLbm = SRAbm	lectin	30 kDa	-	bark	[72,80]
	*Sambucus sieboldiana* (Miq.) Blume ex Graebn.	SSA = SSA-b-1	RIP 2	160 kDa	985 ng/mL; 16.4 nM (A) ^5^	bark	[81,82,83]
		Sieboldin-b = SSA-b-2	RIP 2	59.4 kDa	0.9 ng/mL; 0.015 nM (A) ^5^	bark	[29,83,84]
		SSA-b-3	lectin	34,262 Da	20–30 µg/mL	bark	[83]
		SSA-b-4	lectin	32,333 Da	20–30 µg/mL	bark	[83]
**Aizoaceae**	*Mesembryanthe-mum crystallinum* L.	RIP1	RIP 1	31.6 kDa	n.a. ^4^	leaves	[85]
**Amaranthaceae**	*Amaranthus caudatus* L.	Amaranthin = ACA	lectin	63.5 kDa	-	seeds	[86,87,88]
	*Amaranthus cruentus* L.	ACL	lectin	66 kDa	-	seeds	[89]
	*Amaranthus hypochondriacus* L. [Syn.: *Amaranthus leucocarpus* S. Watson]	*A. leucocarpus* lectin	lectin	45 kDa	-	seeds	[90]
	*Amaranthus mangostanus* L.	Amaramangin	RIP 1	29 kDa	n.a. ^4^	seeds	[91]
	*Amaranthus tricolor* L.	AAP-27	RIP 1	27 kDa	n.a. ^4^	leaves	[92]
**Amaranthaceae**	*Amaranthus viridis* L.	Amaranthin	RIP 1	30 kDa	25 pM	leaves	[93,94]
	*Beta vulgaris* L.	Beetin-27 = BE27	RIP 1	27,592 Da	1.15 ng/mL	leaves	[95,96,97]
		Beetin-29 = BE29	RIP 1	29 kDa	n.a. ^4^	leaves	[95,96,97]
		Betavulgin	RIP 1	30 kDa	n.a. ^4^	seedlings	[98]
	*Celosia argentea* L. [Syn.: *Celosia cristata* L.]	CCP-25	RIP 1	25 kDa	n.a. ^4^	leaves	[99,100]
		CCP-27	RIP 1	27 kDa	25 ng/mL	leaves	[99,100,101]
	*Chenopodium album* L.	CAP30	RIP 1	30 kDa	2.26 pM	leaves	[102,103]
	*Spinacia oleracea* L.	SoRIP1 = BP31	RIP 1	31 kDa	n.a. ^4^	cell cultures	[104,105,106,107]
		SoRIP2	RIP 1 candidate	36 kDa	n.a. ^4^	cell cultures	[106,107]
**Araliaceae**	*Aralia elata* (Miq.) Seem.	Aralin	RIP 2	62 kDa	n.a. ^4^	shoots	[108,109]
	*Panax ginseng* C.A.Mey	Panaxagin	peculiar RIP 1 candidate/RNase	52 kDa	0.28 nM	roots	[110]
	*Panax quinquefolius* L.	Quinqueginsin	peculiar RIP 1 candidate/RNase	53 kDa	0.26 nM	roots	[111]
**Asparagaceae**	*Asparagus officinalis* L.	Asparin 1	RIP 1	30.5 kDa	0.27 nM	seeds	[112,113]
		Asparin 2	RIP 1	29.8 kDa	0.15 nM	seeds	[112,113]
	*Drimia maritima* (L.) Stearn [Syn.: *Charybdis maritima* (L.) Speta]	Charybdin	RIP 1	29 kDa	27.2 nM	bulbs	[114]
	*Muscari armeniacum* Leichtlin ex Baker	Musarmin 1	RIP 1	28,708 Da	7 ng/mL	bulbs	[115]
		Musarmin 2	RIP 1	30,003 Da	9.5 ng/mL	bulbs	[115]
		Musarmin 3	RIP 1	27,626 Da	4 ng/mL	bulbs	[115]
		Musarmin 4	RIP 1	28 kDa	1.4–8.2 ng/mL; 50–280 nM	recomb. ^6^	[116]
	*Polygonatum multiflorum* (L.) All.	PMRIPm	RIP 2	60 kDa	n.a. ^4^	leaves	[117]
		PMRIPt	RIP 2	240 kDa	n.a. ^4^	leaves	[117]
	*Yucca gloriosa* var. *tristis* Carrière [Syn.: *Yucca recurvifolia* Salisb.]	Yucca leaf protein = YLP	RIP 1	23 kDa	n.a. ^4^	leaves	[118,119]
**Basellaceae**	*Basella rubra* L.	*Basella* RIP 2a	RIP 1	30.6 kDa	1.70 ng/mL	seeds	[120]
		*Basella* RIP 2b	RIP 1	31.2 kDa	1.70 ng/mL	seeds	[120]
		*Basella* RIP 3	RIP 1	31.2 kDa	1.66 ng/mL	seeds	[120]
**Caryophyllaceae**	*Agrostemma githago* L.	Agrostin 2	RIP 1	30.6 kDa	0.6 nM	seeds	[121,122]
		Agrostin 5	RIP 1	29.5 kDa	0.47 nM	seeds	[121,122]
		Agrostin 6	RIP 1	29.6 kDa	0.57 nM	seeds	[121,122]
		Agrostin	RIP 1	27 kDa	n.a. ^4^	seeds	[123]
	*Dianthus barbatus* L.	Dianthin 29	RIP 1	29 kDa	1.5 nM	leaves	[124]
	*Dianthus caryophyllus* L.	Dianthin 30	RIP 1	29.5 kDa	9.15 ng/mL; 0.3 nM	leaves	[122,125,126]
		Dianthin 32	RIP 1	31.7 kDa	3.6 ng/mL; 0.12 nM	leaves	[125,126]
	*Dianthus chinensis* L. [Syn.: *Dianthus sinensis* Link]	*D. sinensis* RIP	RIP 1	n.a. ^4^	n.a. ^4^	recomb. ^6^	[127]
	*Gypsophila elegans* M.Bieb.	Gypsophilin	RIP 1	28 kDa	n.a. ^4^	leaves	[128]
	*Silene chalcedonica* (L.) E.H.L.Krause [Syn.: *Lychnis chalcedonica* L.]	Lychnin	RIP 1	26,131 Da	0.17 nM	seeds	[113,129,130]
	*Silene glaucifolia* Lag. [Syn.: *Petrocoptis glaucifolia* (Lag.) Boiss.]	Petroglaucin 1	RIP 1	26.7 kDa	6 ng/mL	whole plants	[131]
		Petroglaucin 2	RIP 1	27.5 kDa	0.7 ng/mL	whole plants	[132]
	*Silene laxipruinosa* Mayol & Rosselló [Syn.: *Petrocoptis grandiflora* Rothm.]	Petrograndin	RIP 1	28.6 kDa	6.6 ng/mL	whole plants	[131]
	*Saponaria ocymoides* L.	Ocymoidin	RIP 1	30.2 kDa	46 pM; 4.8 ng/mL	seeds	[133,134]
	*Saponaria officinalis* L.	Saporin-L1 = SO-L1	RIP 1	31.6 kDa	0.25 nM	leaves	[135,136,137,138]
		Saporin-L2 = SO-L2	RIP 1	31.6 kDa	0.54 nM	leaves	[135]
		Saporin-L3 = SO-L3	RIP 1	n.a. ^4^	n.a. ^4^	leaves	[135]
		Saporin-l = SO-l = SO-4	RIP 1	n.a. ^4^	n.a. ^4^	leaves	[139]
		Saporin-R1 = SO-R1	RIP 1	30.2 kDa	0.86 nM	roots	[135]
		Saporin-R2 = SO-R2	RIP 1	30.9 kDa	0.47 nM	roots	[135]
**Caryophyllaceae**	*Saponaria officinalis* L.	Saporin-R3 = SO-R3	RIP 1	30.9 kDa	0.48 nM	roots	[135]
		SO3a	RIP 1	22.5 kDa	n.a. ^4^	seeds	[140]
		SO3b	RIP 1	19.4 kDa	n.a. ^4^	seeds	[140]
		Saporin-S5 = Saporin 5 = SO-S5	RIP 1	30.5 kDa	0.05 nM; 10.3 ng/mL	seeds	[112,135,141]
		Saporin-S6 = Saporin 6 = SO-6 = SO-S6	RIP 1	28,577 Da	0.06 nM; 0.6 ng/mL	seeds	[112,135,139,141,142,143,144,145]
		Saporin-S8 = SO-S8	RIP 1	n.a. ^4^	n.a. ^4^	seeds	[135]
		Saporin-S9 = Saporin 9 = SO-S9	RIP 1	28,495 Da	0.037 nM	seeds	[112,122,135,146]
		SAP-C	RIP 1	28.5 kDa	125 pM	recomb. ^6^	[147]
		SAP-S	RIP 1	28,560 Da	12 pM	seeds	[147]
	*Myosoton aquaticum* (L.) Moench [Syn.: *Stellaria aquatica* (L.) Scop.]	Stellarin	RIP 1	25 kDa	0.04 nM	leaves	[148]
	*Stellaria media* (L.) Vill.	RIP Q3	RIP 1	28.2 kDa	n.a. ^4^	recomb. ^6^	[149]
	*Vaccaria hispanica* (Mill.) Rauschert [Syn.: *Vaccaria pyramidata* Medik.]	Pyramidatin	RIP 1	28.0 kDa	89 pM; 3.6 ng/mL	seeds	[133]
**Cucurbitaceae**	*Benincasa hispida* (Thunb.) Cogn.	Hispin	RIP 1	21 kDa	165 pM	seeds	[150]
		α-benincasin	sRIP 1	12 kDa	20 pM; 0.22 ng/mL	seeds	[151]
		β-benincasin	sRIP 1	12 kDa	320 pM; 3.4 ng/mL	seeds	[151]
	*Bryonia cretica* subsp. *dioica* (Jacq.) Tutin. [Syn.: *Bryonia dioica* L.]	Bryodin 1 = BD1	RIP 1	29 kDa	0.12 nM; 3.6 ng/mL; 7 pM	roots	[152,153]
		Bryodin 2	RIP 1	27 kDa	9 pM	roots	[153]
		Bryodin-L	RIP 1	28.8 kDa	0.09 nM	leaves	[113]
		Bryodin-R	RIP 1	n.a. ^4^	n.a. ^4^	seeds	[154,155]
		BDA	lectin/RIP 2 like	61 kDa	>1500 nm	roots	[73,156]
**Cucurbitaceae**	*Citrullus colocynthis* (L.) Schrad.	Colocin 1	RIP 1	26.3 kDa	0.04 nM	seeds	[113]
		Colocin 2	RIP 1	26.3 kDa	0.13 nM	seeds	[113]
	*Cucurbita foetidissima* Kunth	Foetidissimin	peculiar RIP 2	63 kDa	25.9 nM	roots	[157]
		Foetidissimin II	RIP 2	61 kDa	251.6 nM	roots	[158]
	*Cucumis ficifolius* A.Rich. [Syn.: *Cucumis figarei* Delile ex Naudin]	*Cucumis figarei* RIP = CF-RIP	RIP 1 candidate	n.a. ^4^	n.a. ^4^	recomb. ^6^	[159]
	*Cucurbita maxima* Duchesne	Cucurmoschin	sRIP 1 candidate	9 kDa	1.2 µM	seeds	[160]
	*Cucurbita moschata* Duchesne [Syn.: *Cucurbita moschata* (Duchesne ex Lam.) Duchesne ex Poir.]	Cucurmosin	RIP 1	27–28 kDa	n.a. ^4^	sarcocarp	[161,162,163]
		Cucurmosin 2	RIP 1	27,183 Da	n.a. ^4^	sarcocarp	[164,165]
		*C. moschata* RIP	RIP 1	30,665 Da	0.035 nM; 1.08 ng/mL	skinned fruit	[155]
		Moschatin	RIP 1	29 kDa	0.26 nM	seeds	[166]
		PRIP 1	RIP 1	31 kDa	0.82 nM	leaves	[167]
		PRIP 2	RIP 1	30.5 kDa	0.79 nM	leaves	[167]
		α-moschin	sRIP 1 candidate	12 kDa	17 µM	seeds	[168]
		β-moschin	sRIP 1 candidate	12 kDa	300 nM	seeds	[168]
	*Cucurbita pepo* L.	Pepocin	RIP 1	26 kDa	15.4 pM	sarcocarp	[169]
	*Cucurbita pepo* var. *texana* (Scheele) D.S.Decker [Syn.: *Cucurbita texana* (Scheele) A. Gray]	Texanin	RIP 1	29.7 kDa	n.a. ^4^	fruits	[158]
	*Gynostemma pentaphyllum* (Thunb.) Makino	Gynostemmin	RIP 1	27 kDa	n.a. ^4^	leaves and stems	[170]
	*Lagenaria siceraria* (Molina) Standl.	Lagenin	RIP 1 candidate	20 kDa	0.21 nM	seeds	[171]
	*Luffa acutangula* (L.) Roxb.	Luffaculin-1	RIP 1	28 kDa	3.6 ng/mL; 124 pM	seeds	[172,173]
		Luffaculin-2	RIP 1	28 kDa	n.a. ^4^	seeds	[173]
		Luffangulin	sRIP 1	5.6 kDa	3.5 nM	seeds	[174]
		*Luffa acutangula* fruit lectin	lectin	48 kDa	-	fruits	[175]
**Cucurbitaceae**	*Luffa cylindrica* (L.) M.Roem [Syn.: *Luffa aegyptiaca* Mill.]	Luffin	RIP 1	26 kDa	0.42 ng/mL	seeds	[176]
		Luffin-a	RIP 1	27,021 Da	1.64 ng/mL	seeds	[177,178]
		Luffin-b	RIP 1	27,275 Da	0.84 ng/mL	seeds	[177,178]
		α-luffin	RIP 1	28 kDa	10 ng/mL; 34.1 pM (recomb. ^6^)	seeds	[179,180,181]
		β-luffin	RIP 1	29 kDa	50 ng/mL	seeds	[180,182]
		LRIP	RIP 1	30 kDa	8 pM	seeds	[183]
		Luffacylin	sRIP 1	7.8 kDa	0.14 nM	seeds	[184]
		Luffin P1	sRIP 1	5226.1 Da	0.88 nM	seeds	[185]
		Luffin-S	sRIP 1 candidate	10 kDa	0.34 nM	seeds	[186]
		LuffinS(1)	sRIP 1 candidate	8 kDa	130 nM	seeds	[187]
		LuffinS(2) = luffin S2	sRIP 1 candidate	7.8 kDa	10 nM	seeds	[187,188]
		LuffinS(3)	sRIP 1 candidate	8 kDa	630 nM	seeds	[187]
	*Marah oreganus* (Torr. & A. Gray) Howell	MOR-I	RIP 1	27,989 Da	0.063 nM	seeds	[189]
		MOR-II	RIP 1	27,632 Da	0.071 nM	seeds	[189]
	*Momordica balsamina* L.	Balsamin	RIP 1	28.6 kDa	90.6 ng/mL	seeds	[190]
		MbRIP-1	RIP 1	30 kDa	n.a. ^4^	seeds	[191,192]
		Momordin II	RIP 1	n.a. ^4^	n.a. ^4^	recomb. ^6^	[193]
	*Momordica charantia* L.	MAP 30	RIP 1	30 kDa	3.3 nM	seeds and fruits	[194,195]
		α-momorcharin = α-MC = α-MMC	RIP 1	28,625–28,795 Da	0.23 nM	seeds	[196,197,198,199,200,201,202,203,204]
		β-momorcharin = β-MC = β-MMC	RIP 1	29,074–29,076 Da	0.19 nM	seeds	[196,197,198,200,201,202,203]
		γ-momorcharin = γ-MMC	sRIP 1	11.5 kDa	55 nM	seeds	[205]
		δ-momorcharin = δ-MMC	RIP 1	30 kDa	0.15 nM	seeds	[203]
		ε-momorcharin	RIP 1 candidate	24 kDa	170 nM	fruits	[203]
		Momordin	RIP 1	31 kDa	n.a. ^4^	seeds	[206]
		Momordin *= Momordica charantia* inhibitor	RIP 1	23–24 kDa	1.8 ng/mL	seeds	[207,208,209,210,211,212]
		Momordin II	RIP 1	n.a. ^4^	n.a. ^4^	seeds	[213]
**Cucurbitaceae**	*Momordica charantia* L.	Momordin-a	RIP 1	29.4 kDa	n.a. ^4^	seeds	[214,215]
		Momordin-b	RIP 1	29.4 kDa	n.a. ^4^	seeds	[214]
		Charantin	sRIP 1	9.7 kDa	400 nM	seeds	[216]
		MCL *= M. charantia* lectin	lectin	12.4 kDa	-	seeds	[217]
		MCL *= Momordica charantia* seed lectin = *Momordica charantia* lectin	RIP 2	115–124 kDa	1.74 µg/mL; 5 µg/mL	seeds	[207,218,219,220]
		MCL1	RIP 2	60,993 Da	1.9 nM	seeds	[221]
		anti-H Lectin	lectin	150 kDa	-	seeds	[222]
		Momordica agglutinin	lectin	30 kDa	-	seeds	[223]
		Momordin	lectin	22–23 kDa	-	seeds	[223]
		protein fraction 1	lectin	49 kDa	-	seeds	[224]
		protein fraction 2	lectin	49 kDa	-	seeds	[224]
	*Momordica cochinchinensis* Spreng.	Cochinin B	RIP 1	28 kDa	0.36 nM	seeds	[225]
		Momorcochin	RIP 1	32 kDa	n.a. ^4^	tubers	[200,226]
		Momorcochin-S	RIP 1	30 kDa	0.12 nM	seeds	[225,227]
	*Siraitia grosvenorii* (Swingle) C.Jeffrey ex A.M.Lu & Zhi Y.Zhang [Syn.: *Momordica grosvenorii* Swingle]	Momorgrosvin	RIP 1	27.7 kDa	0.3 nM	seeds	[228]
	*Sechium edule* (Jacq.) Sw.	Sechiumin	RIP 1	27 kDa	0.7 nM	seeds	[229]
		*Sechium edule* fruit lectin	lectin	44 kDa	-	fruits	[230]
	*Trichosanthes anguina* L.	Trichoanguin	RIP 1	35 kDa	0.08 nM	seeds	[231]
		SGSL	lectin/RIP 2 like	62 kDa	n.a. ^4^	seeds	[232,233,234]
	*Trichosanthes cordata* Roxb.	TCA-I	lectin	59 kDa	n.a. ^4^	seeds	[235]
		TCA-II	lectin	52 kDa	n.a. ^4^	seeds	[235]
**Cucurbitaceae**	*Trichosanthes cucumerina* L.	TCSL	lectin/RIP 2 candidate	69 kDa	n.a. ^4^	seeds	[236]
	*Trichosanthes cucumeroides* (Ser.) Maxim.	β-trichosanthin = β-TCS	RIP 1	28 kDa	2.8 ng/mL; 0.1 nM	root tubers	[200,237,238]
	*Trichosanthes kirilowii* Maxim.	α-kirilowin	RIP 1	28.8 kDa	1.2-1.8 ng/mL; 0.044–0.066 mM	seeds	[239]
		β-kirilowin	RIP 1	27.5 kDa	1.8 ng/mL	seeds	[240]
		TAP 29	RIP 1	29 kDa	3.7 nM	root tubers	[241,242]
		TK-35	RIP 1	35,117 Da	2.45 nM	cell cultures	[243]
		Trichobitacin	RIP 1	27,228 Da	n.a. ^4^	root tubers	[244,245,246]
		Trichokirin	RIP 1	27 kDa	0.06–0.13 nM	seeds	[247]
		Trichomislin = TCM	RIP 1	27,211 Da	2.26 nM	recomb. ^6^	[248]
		Trichosanthin = Trichosanthes antiviral protein = TAP = TCS = α-trichosanthin = α-TCS = GLQ223	RIP 1	26–28 kDa	6.1 ng/mL; 0.23 nM; 0.36 ng/mL; 1.31 nM	root tubers	[198,200,238,248,249,250,251,252,253,254,255,256]
		Trichosanthin	RIP 1	25 kDa	n.a. ^4^	root tubers	[257]
		β-trichosanthin = β-TCS	RIP 1	26 kDa	7 ng/mL	root tubers	[255]
		γ-trichosanthin = γ-TCS	RIP 1	26 kDa	12 ng/mL	root tubers	[255]
		Trichokirin S1	sRIP 1	11,426 Da	0.7 nM	seeds	[258]
		S-Trichokirin	sRIP 1	8 kDa	115 pM	seeds	[259]
		Trichosanthrip	sRIP 1	10,964 Da	1.6 ng/mL	seeds	[256]
		TKL-1 = *Trichosanthes kirilowii* lectin-1	lectin/RIP 2 candidate	60 kDa	n.a. ^4^	root tubers	[260,261]
		TK-I	lectin	n.a. ^4^	-	root tubers	[262,263]
		TK-II	lectin	n.a. ^4^	-	root tubers	[262,263]
		TK-III	lectin	n.a. ^4^	-	root tubers	[262,263]
		*Trichosanthes kirilowii* lectin	lectin	57 kDa	-	seeds	[264]
**Cucurbitaceae**	*Trichosanthes kirilowii* Maximoviczvar. *japonica* (Miquel) Kitamura	Karasurin-A	RIP 1	27,215 Da	0.1–0.3 ng/mL	root tubers	[265,266,267,268]
		Karasurin-B	RIP 1	27,214 Da	0.1–0.3 ng/mL	root tubers	[267]
		Karasurin-C	RIP 1	27,401 Da	0.1–0.3 ng/mL	root tubers	[267]
	*Trichosanthes lepiniate*	Trichomaglin	RIP 1	24,673 Da	10.1 nM	root tuber	[269]
	*Trichosanthes dioica* Roxb.	TDSL	lectin/RIP 2 candidate	55 kDa	n.a. ^4^	seeds	[270]
	*Trichosanthes* sp. Bac Kan 8-98	Trichobakin	RIP 1	27 kDa	3.5 pM	leaves	[271]
**Cupressaceae**	*Thuja occidentalis* L.	Arborvitae RIP	RIP candidate	n.a. ^4^	n.a. ^4^	seeds	[272]
**Euphorbiaceae**	*Croton tiglium* L.	Crotin I	RIP 1 candidate	40 kDa	n.a. ^4^	seeds	[273,274,275]
		Crotin 2	RIP 1	n.a. ^4^	n.a. ^4^	seeds	[276,277,278]
	*Euphorbia characias* L.	*E. characias* lectin	lectin	80 kDa	-	latex	[279]
	*Suregada multiflora* (A.Juss.) Baill. [Syn.: *Gelonium multiflorum* A.Juss.]	Gelonin = GAP 31	RIP 1	30–31 kDa	0.406 ng/mL; 0.32 nM	seeds	[126,280,281,282,283]
	*Hura Crepitans* L.	*Hura crepitans* RIP	RIP 1	28 kDa	n.a. ^4^	latex, leaves	[27,112]
		*Hura crepitans* RIP-5	RIP 1	n.a. ^4^	n.a. ^4^	latex	[284]
		*Hura crepitans* latex lectin	RIP 2	112 kDa	-	latex	[279]
		Crepitin	lectin	n.a. ^4^	n.a. ^4^	latex	[285,286]
		Hurin	lectin	70 kDa	-	seeds	[287,288]
		*Hura crepitans* seed lectin	lectin	120 kDa	-	seeds	[286]
	*Jatropha curcas* L.	Curcin	RIP 1	28.2 kDa	0.42 nM	seeds	[273,289]
		Curcin 2	RIP 1	30.1 kDa	n.a. ^4^	recomb. ^6^	[290,291]
		Curcin-L	RIP 1	32 kDa	4 µg/mL	leaves	[292,293]
		Jc-SCRIP	RIP 1	38,938 Da	n.a. ^4^	seed coat	[294]
	*Manihot palmata* Müll. Arg.	Mapalmin	RIP 1	32.3 kDa	0.05 nM	seeds	[113]
**Euphorbiaceae**	*Manihot esculenta* Crantz. [Syn.: *Manihot utilissima* Pohl]	Manutin 1	RIP 1	n.a. ^4^	0.05 nM	seeds	[284,295]
		Manutin 2	RIP 1	n.a. ^4^	0.12 nM	seeds	[295]
	*Ricinus communis* L.	Ricin = crystalline Ricin = Ricin D	RIP 2	62.8 kDa	0.14 nM (A) ^5^; 814 pM; 5.5 ng/mL	seeds	[59,281,296,297,298,299,300,301,302,303,304,305,306,307,308,309]
		Ricin E	RIP 2	64 kDa	n.a. ^4^	seeds	[310,311,312]
		RCA = *Ricinus communis* agglutinin = RCA_I_ = RCA_120_ = *R. communis* hemagglutinin = RCB-PHA I	RIP 2	118–130 kDa	n.a. ^4^	seeds	[303,313,314,315,316,317,318,319,320,321]
		RCA_II_ = RCA_60_ = RCB-PHA II	RIP 2	60 kDa	n.a. ^4^	seeds	[313,314,316,317]
	*Ricinus communis*, USA	Ricin 1	RIP 2	66 kDa	n.a. ^4^	seeds	[303,322]
		Ricin 2	RIP 2	66 kDa	n.a. ^4^	seeds	[303,322]
		Ricin 3	RIP 2	66 kDa	n.a. ^4^	seeds	[303,322]
	*Ricinus communis*, India	Ricin I	RIP 2	64 kDa	n.a. ^4^	seeds	[322,323]
		Ricin II	RIP 2	64 kDa	n.a. ^4^	seeds	[322,323]
		Ricin III	RIP 2	64 kDa	n.a. ^4^	seeds	[322,323]
	*Ricinus sanguienus,* France	Ricin_11_	RIP 2	57,805 Da	n.a. ^4^	seeds	[322,324]
		Ricin_12_	RIP 2	62,163 Da	n.a. ^4^	seeds	[322,324]
		Ricin_2_	RIP 2	63,116 Da	n.a. ^4^	seeds	[322,324]
**Fabaceae**	*Abrus precatorius* L.	Abrin	RIP 2	260 kDa	0.5 nM (A) ^5^	seeds	[29,307,315,323,325,326,327,328,329,330]
		Abrin-a = Abrin C = Abrin-III	RIP 2	63–65.5 kDa	60 pM (A) ^5^	seeds	[331,332,333,334,335,336,337,338,339,340]
		Abrin-b	RIP 2	67 kDa	n.a. ^4^	seeds	[333,334,335,338]
		Abrin-c = Abrin A = Abrin-I	RIP 2	60.1–62.5 kDa	n.a. ^4^	seeds	[331,332,334,335,336,337]
		Abrin-d	RIP 2	67 kDa	n.a. ^4^	seeds	[334,335,338]
		Abrin-II	RIP 2	63 kDa	n.a. ^4^	seeds	[337]
**Fabaceae**	*Abrus precatorius* L.	APA = *Abrus precatorius* agglutinin = Abrus lectin = AAG	RIP 2	126–134 kDa	3.5 nM	seeds	[315,334,341,342,343,344,345]
		APA-I	RIP 2	130 kDa	n.a. ^4^	seeds	[337,346]
		APA-II	RIP 2	128 kDa	n.a. ^4^	seeds	[337]
	*Abrus pulchellus* Thwaites	Pulchellin	RIP 2	62 kDa	n.a. ^4^	seeds	[347,348,349]
		Pulchellin PI	RIP 2	61.5–63 kDa	n.a. ^4^	seeds	[350]
		Pulchellin PII	RIP 2	61.5–63 kDa	n.a. ^4^	seeds	[350]
		Pulchellin PIII	RIP 2	61.5–63 kDa	n.a. ^4^	seeds	[350]
	*Pisum sativum* subsp. *sativum* L. [Syn.: *Pisum sativum* var. *arvense* (L.) Poir.]	α-pisavin	RIP 1	20.5 kDa	0.5 nM	seeds	[351]
		β-pisavin	RIP 1	18.7 kDa	0.5 nM	seeds	[351]
	*Pisum sativum* var. *macrocarpon*	Sativin	RIP 1 candidate	38 kDa	14 µM	legumes	[352]
**Iridaceae**	*Iris hollandica* var. Professor Blaauw	IrisRIP = IRIP	RIP 1	28 kDa	0.1–0.16 nM	bulbs	[353,354]
		IrisRIP.A1	RIP 1	29 kDa	0.16 nM	bulbs	[353]
		IrisRIP.A2	RIP 1	29 kDa	0.12 nM	bulbs	[353]
		IrisRIP.A3	RIP 1	29 kDa	0.10 nM	bulbs	[353]
		IRA	RIP 2	60.4 kDa	n.a. ^4^	bulbs	[355]
		IRAb	RIP 2	65 kDa	n.a. ^4^	bulbs	[356,357]
		IRAr	RIP 2	65 kDa	n.a. ^4^	bulbs	[356]
**Lamiaceae**	*Clerodendrum aculeatum* (L.) Schltdl.	CA-SRI	RIP 1 candidate	34 kDa	<0.01 nM	leaves	[358,359]
	*Clerodendrum inerme* (L.) Gaertn.	CIP-29	RIP 1	29 kDa	0.548 nM; 16 ng/mL	leaves	[360,361]
		CIP-34	RIP 1 candidate	34 kDa	87.4 nM; 3 µg/mL	leaves	[360,361]
	*Leonurus japonicus* Houtt.	Leonurin	RIP candidate	n.a. ^4^	n.a. ^4^	seeds	[362]
**Lauraceae**	*Cinnamomum bodinieri* H. Lév.	Bodinierin	RIP 2	65 kDa	1.2 nM (A) ^5^	kernel	[363]
**Lauraceae**	*Cinnamomum camphora* (L.) J.Presl	Camphorin	RIP 1	23 kDa	0.098 nM	seeds	[364,365]
		Cinnamomin	RIP 2	61 kDa	9.7 nM (A) ^5^	seeds	[364,365,366,367]
		Cinnamomin 1	RIP 2	61 kDa	n.a. ^4^	seeds	[364]
		Cinnamomin 2	RIP 2	n.a. ^4^	n.a. ^4^	seeds	[364]
		Cinnamomin 3	RIP 2	n.a. ^4^	n.a. ^4^	seeds	[364]
		Cinphorin	sRIP 2	46 kDa	1.2 nM	seeds	[367,368]
	*Cinnamomum parthenoxylon* (Jack) Meisn. [Syn.: *Cinnamomum porrectum* (Roxb.) Kosterm.]	Porrectin	RIP 2	64.5 kDa	0.11 µM	seeds	[369]
**Malvaceae**	*Abelmoschus esculentus* (L.) Moench	Abelesculin	RIP 1	30 kDa	n.a. ^4^	seeds	[370]
**Nyctaginaceae**	*Boerhaavia diffusa* L.	*Boerhaavia* inhibitor	RIP 1 candidate	16–20 kDa	n.a. ^4^	roots	[371,372,373]
	*Bougainvillea spectabilis* Willd.	BAP I	RIP 1	28 kDa	n.a. ^4^	roots	[374]
		Bouganin = *Bougainvillea* RIP I	RIP 1	26.2 kDa	10.5 ng/mL	leaves	[120,375]
	*Bougainvillea* × *buttiana cv.* Enid Lancester	BBP-24	RIP 1	24 kDa	n.a. ^4^	leaves	[376,377]
		BBP-28	RIP 1	28 kDa	n.a. ^4^	leaves	[376,377]
	*Bougainvillea* × *buttiana* *cv.* Mahara	BBAP1	RIP 1	35.49 kDa	n.a. ^4^	leaves	[378,379]
	*Mirabilis expansa* (Ruiz & Pav.) Standl.	ME1	RIP 1	29,208 Da	n.a. ^4^	roots	[380,381]
		ME2	RIP 1	27 kDa	n.a. ^4^	roots	[380]
	*Mirabilis jalapa* L.	MAP	RIP 1	27,788 Da	5.4 ng/mL	roots/seeds	[373,382,383]
		MAP-2	RIP 1	30,412 Da	41.4 ng/mL	seeds	[383]
		MAP-3	RIP 1	29,771 Da	13.3 ng/mL	seeds	[383]
		MAP-4	RIP 1	29,339 Da	15.3 ng/mL	seeds and leaves	[383]
		MAP-S	RIP 1	27,789 Da	n.a. ^4^	seeds	[146]
**Olacaceae**	*Malania oleifera* Chun & S. K. Lee	Malanin	lectin/RIP 2 candidate	61875 Da	n.a. ^4^	seeds	[384]
**Olacaceae**	*Ximenia americana* L.	Riproximin = Rpx	RIP 2	56 kDa	n.a. ^4^	fruit kernels	[385,386]
		Rpx-I	RIP 2	50 kDa	n.a. ^4^	fruit kernels	[386]
		Rpx-II	RIP 2	53 kDa	n.a. ^4^	fruit kernels	[386]
**Passifloraceae**	*Adenia digitata* (Harv.) Engl.	Modeccin = Modeccin 4B	RIP 2	57–63 kDa	4 µg/mL; 2.52 µg/mL; 66 ng/mL (A) ^5^	roots	[387,388,389,390]
		Modeccin 6B	RIP 2	57 kDa	0.31 µg/mL	roots	[390]
	*Adenia ellenbeckii* Harms	*A. ellenbeckii* lectin	RIP 2 candidate	60 kDa	10.1 µg/mL; 1.2 µg/mL (A) ^5^	caudex	[45]
	*Adenia fruticosa* Burtt Davy	*A. fruticosa* lectin	lectin	30 kDa	>100 µg/mL	caudex	[45]
	*Adenia glauca* Schinz	*A. glauca* lectin	RIP 2 candidate	n.a. ^4^	>10 µg/mL; >5 µg/mL (A) ^5^	caudex	[45]
	*Adenia goetzei* Harms (unresolved name)	*A. goetzei* lectin	RIP 2	60 kDa	55.1 µg/mL; 0.7 µg/mL (A) ^5^	caudex	[45]
	*Adenia keramanthus* Harms	*A. keramanthus* lectin	RIP 2 candidate	60–65 kDa	10.0 µg/mL; 1.1 µg/mL (A) ^5^	caudex	[45]
	*Adenia lanceolata* Engl.	Lanceolin	RIP 2	60 kDa	5.2 µg/mL; 1.1 µg/mL (A) ^5^	caudex	[45,391,392]
	*Adenia racemosa* W. J. de Wilde	*A. racemosa* lectin	lectin	30 kDa	>400 µg/mL	caudex	[45]
	*Adenia spinosa* Burtt Davy	*A. spinosa* lectin	RIP 2 candidate	n.a. ^4^	4.7 µg/mL; 0.8 µg/mL (A) ^5^	caudex	[45]
	*Adenia stenodactyla* Harms	Stenodactylin	RIP 2	60 kDa	5.6 µg/mL; 0.5 µg/mL (A) ^5^	caudex	[45,391,392]
	*Adenia venenata* Forssk.	*A. venenata* lectin	RIP 2 candidate	60 kDa	2.4 µg/mL; 0.4 µg/mL (A) ^5^	caudex	[45]
	*Adenia volkensii* Harms	Volkensin	RIP 2	62 kDa	5 µg/mL; 84 nM; 0.37 nM (A) ^5^ 22 ng/mL (A) ^5^; 7.5 µg/mL; 0.66 µg/mL (A) ^5^	roots	[45,393,394,395]
**Phytolaccaceae**	*Phytolacca americana* L.	α-PAP	RIP 1	33,068 kDa	n.a. ^4^	recomb. ^6^	[396,397]
		PAP = *Phytolacca americana* protein = pokeweed antiviral protein	RIP 1	29–30 kDa	0.29 nM	leaves	[29,398,399,400,401,402,403]
		PAP-I	RIP 1	30 kDa	2 pM	spring leaves	[404]
		PAP-II	RIP 1	30–31 kDa	4 pM	early summer leaves	[399,400,404,405]
		PAP-III	RIP 1	30 kDa	3 pM	late summer leaves	[404]
		PAP-C	RIP 1	29 kDa	0.062 nM; 2 ng/mL	cell cultures	[406]
		PAP-H	RIP 1	29.5 kDa	n.a. ^4^	hairy roots	[407]
		PAP-R	RIP 1	29.8 kDa	0.05 nM	roots	[113]
		PAP-S	RIP 1	30 kDa	36-83 nM; 1.09–2.5 ng/mL	seeds	[399,408]
		PAP-S1	RIP 1	n.a. ^4^	n.a. ^4^	recomb. ^6^	[397]
		PAP-S2	RIP 1	n.a. ^4^	n.a. ^4^	recomb. ^6^	[397]
	*Phytolacca dioica* L.	Diocin 1	RIP 1	30,047 Da	19.74 ng/mL; 0.658 nM	leaves of young plants	[409]
		Diocin 2	RIP 1	29,910 Da	6.85 ng/mL; 0.229 nM	leaves of young plants	[409]
		PD-L1	RIP 1	32,715 Da	102 pM; 3.32 ng/mL; 8.5 pM	leaves	[410,411]
		PD-L2	RIP 1	31,542 Da	110 pM; 3.46 ng/mL	leaves	[410,412]
		PD-L3	RIP 1	30,356 Da	228 pM; 6.93 ng/mL	leaves	[410,412]
		PD-L4	RIP 1	29185 Da	134 pM; 3.92 ng/mL	leaves	[410,413]
		PD-S1	RIP 1	30.9 kDa	0.12 nM	seeds	[414]
		PD-S2	RIP 1	29,586 Da	0.06 nM	seeds	[414,415]
		PD-S3	RIP 1	32 kDa	0.08 nM	seeds	[414]
**Phytolaccaceae**	*Phytolacca dodecandra* L’Hér.	Dodecandrin	RIP 1	29 kDa	n.a. ^4^	leaves	[416,417]
		Dodecandrin C	RIP 1	31–32 kDa	n.a. ^4^	cell cultures	[417]
	*Phytolacca heterotepala* H. Walter	Heterotepalin 4	RIP 1	29,326 Da	82 pM	leaves	[418]
		Heterotepalin 5b	RIP 1	30,477 Da	52 pM	leaves	[418]
	*Phytolacca insularis* Nakai	Insularin = PIP = *Phytolacca insularis* antiviral protein	RIP 1	31 kDa	n.a. ^4^	recomb. ^6^	[7,419]
		PIP2 = *P. insularis* antiviral protein 2	RIP 1	29.6 kDa	0.04 nM	recomb. ^6^	[420]
**Poaceae**	*Hordeum vulgare* L.	Barley toxin = Barley translation inhibitor = Barley Protein Synthesis Inhibitor = BPSI = RIP 30	RIP 1	30 kDa	0.47 nM	seeds	[281,421,422,423,424]
		Barley toxin I = Barley translation inhibitor I	RIP 1	30 kDa	25 ng/mL	seeds	[422]
		Barley toxin II = Barley translation inhibitor II = Barley Protein Synthesis Inhibitor II = BPSI II	RIP 1	29,836 Da	25 ng/mL	seeds	[281,421,422,425]
		Barley toxin III = Barley translation inhibitor III	RIP 1	30 kDa	15 ng/mL	seeds	[281,422]
		JIP60	RIP 3/peculiar RIP 1	60 kDa	n.a. ^4^	recomb. ^6^	[5,426]
**Poaceae**	*Oryza sativa* L.	*Oryza sativa* RIP	RIP 1	27 kDa	n.a. ^4^	recomb. ^6^	[427]
	*Secale cereale* L.	RPSI	RIP 1	30,171 Da	0.42 µg/mL	seeds	[421,428]
	*Triticum aestivum* L.	Tritin	RIP 1	30 kDa	n.a. ^4^	germ	[421,429,430,431]
		Tritin 1	RIP 1	30 kDa	250 ng/mL	whole wheat	[432]
		Tritin 2	RIP 1	30 kDa	250 ng/mL	whole wheat	[432]
		Tritin 3	RIP 1	30 kDa	250 ng/mL	whole wheat	[432]
		Tritin-S	RIP 1	32.1–32.8 kDa	n.a. ^4^	seeds	[433]
		Tritin-L	RIP 1	37.0–37.9 kDa	n.a. ^4^	leaves	[433]
	*Zea mays* L.	b-32 = maize RIP = maize proRIP1	RIP 3/peculiar RIP 1	34 kDa	28–60 pM; 0.7–1.5 ng/mL; 0.065 nM	seeds	[4,434,435,436,437,438]
		Maize proRIP2	RIP 3/peculiar RIP 1	31.1 kDa	n.a. ^4^	recomb. ^6^	[436,437]
**Ranunculaceae**	*Eranthis hyemalis* (L.) Salisb.	EHL	RIP 2	62 kDa	n.a. ^4^	root tubers	[439,440]
**Santalaceae**	*Phoradendron californicum* Nutt.	PCL	RIP 2	69 kDa	n.a. ^4^	n.n	[441]
	*Viscum album* L. (Himalayan mistletoe)	HmRip	RIP 2	65 kDa	n.a. ^4^	leaves	[442,443,444]
		HmRip 1	RIP 2	65 kDa	n.a. ^4^	leaves	[442,443,444]
		HmRip 2	RIP 2	65 kDa	n.a. ^4^	leaves	[442,443,444]
		HmRip 3	RIP 2	65 kDa	n.a. ^4^	leaves	[442,443,444]
		HmRip 4	RIP 2	65 kDa	n.a. ^4^	leaves	[442,443,444]
	*Viscum album* L. (European mistletoe)	ML-I = Mistletoe lectin I = Viscumin = Eu-ML = EML-1 = VAA-I	RIP 2	115–125 kDa	2.6 µg/mL; 0.21 µg/mL (A) ^5^; 3.7 pM (A) ^5^	leaves	[234,445,446,447,448,449,450,451,452,453,454]
		ML-II = Mistletoe lectin II = VAA-II	RIP 2	60–64 kDa	n.a. ^4^	leaves	[448,450,451,452]
		ML-III = Mistletoe lectin III = VAA-III	RIP 2	50–61 kDa	n.a. ^4^	leaves	[448,450,451,452]
**Santalaceae**	*Viscum articulatum Burm. f.*	Articulatin-D	RIP 2	66 kDa	n.a. ^4^	whole plant	[455]
	*Viscum coloratum (*Kom.) Nakai [Syn.: *Viscum album* subsp. *coloratum* Kom.]	KML	RIP 2	n.a. ^4^	n.a. ^4^	leaves	[456]
		KML-C	RIP 2	59.5 kDa	n.a. ^4^	leaves	[454,457]
		KML-IIL	RIP 2	60 kDa	n.a. ^4^	leaves	[457]
		KML-IIU	RIP 2	64 kDa	n.a. ^4^	leaves	[457]
		VCA	RIP 2	60 kDa	n.a. ^4^	leaves	[458,459]
**Solanaceae**	*Nicotiana tabacum* L.	CIP31	RIP-like protein	31 kDa	n.a. ^4^	leaves	[460]
		TRIP	RIP 1 candidate	26 kDa	100 ng/mL	leaves	[461]
**Thymelaeaceae**	*Phaleria macrocarpa* (Scheff.) Boerl.	*P. macrocarpa* RIP	RIP candidate	n.a. ^4^	n.a. ^4^	seeds	[462]

^1^ For the botanical name of the plant species we chose the current accepted name from www.theplantlist.org. In some cases, there is also given a synonym, because the protein/RIP is derived from that synonym that is given in the corresponding reference; ^2^ For the values of molecular weight (Mw) we listed the latest values from the native unreduced proteins obtained from gel filtration or from SDS-PAGE. If there were too many different values from different authors, we listed a range. We listed the exact value obtained from MALDI-TOF, ESI-TOF or Q-TOF, if this was available; ^3^ IC_50_ is the half minimal inhibitory concentration (50%) of the protein, which inhibits translation from a cell free system using rabbit reticulocyte lysate. For the IC_50_ values, we listed the values of the molar mass or concentration in mg/mL. In some cases, there were many different values from different laboratories that led us to list a range; ^4^ n.a. = not available; in the case of IC_50_ values there are several reasons for n.a.: 1. The translation-inhibitory assay was not performed; 2. The translation-inhibitory assay was performed by using another system than the cell free system with rabbit reticulocyte lysate, e.g., cancer cells; 3. The IC_50_ values were specified with another unit, e.g., mg/kg; ^5^ (A) = A-chain; the IC_50_ value followed by (A) is for the reduced type 2 RIP; ^6^ recomb. = recombinant; Proteins obtained through biotechnological procedures.

## 3. Exceptions Prove the Rule

To be classified as “classical” type 1 or type 2 RIP, a protein needs both the structure and the *N*-glycosidase activity including the conserved amino acid residues, which are believed to be present in all RIPs, of the putative active site region [94,101,463]. This active site region is also known as shiga/ricin toxic domain [106]. Beside the peculiar type 1 RIPs, b-32 and JIP60, there is a certain amount of other proteins that cannot be grouped into the classical type 1 or type 2 RIPs, because of structural and functional differences.

### 3.1. Small RIPs

First of all, there are “small type 1 RIPs” (sRIP 1; Table 2), which are single chain proteins exhibiting *N*-glycosidase activity with a smaller molecular weight than the classical type 1 RIPs. Interestingly, all known small type 1 RIPs are synthesized by plants belonging to the family Cucurbitaceae. α-luffin and β-luffin from *Luffa cylindrica* indeed have the same size as the other small type 1 RIPs, but because of their lower toxicity of 17 µM and 300 nM, respectively, and due to the unknown mechanism of action, they are classified as “small type 1 RIP candidates” (Table 3). Also luffin-S, luffinS(1), luffinS(2), and luffinS(3) have similar sizes as the other small type 1 RIPs and all of them inhibit protein synthesis in a cell-free system, but it was not analyzed, whether the translation-inhibitory is due to the *N*-glycosidase activity or not. In addition, a different mechanism of action was found for luffin-S [186]. For this reason, the luffinSs are considered as small type 1 RIP candidates (Table 3). Another small type 1 RIP candidate is cucurmoschin that was designated as an antifungal protein by the authors [160]. Cucurmoschin indeed inhibits protein synthesis in a cell-free system, but there was no homology with other type 1 RIPs or small type 1 RIPs concerning the amino acid sequence specified, but the fact that the *N*-glycosidase activity was neither verified nor excluded led us to the decision to classify cucurmoschin as a small type 1 RIP candidate. Lagenin, α-pisavin and β-pisavin have molecular weights of 20 kDa, 20.5 kDa and 18.7 kDa, respectively; thus, they differ from the classical type 1 RIPs as well as from the small type 1 RIPs. Lagenin inhibits cell-free translation in a rabbit reticulocyte system, but it was not clarified whether this is due to the *N*-glycosidase activity [171]. Because the size of lagenin is closer to the classical type 1 RIPs than to the biggest known small type 1 RIPs (α-benincasin and β-benincasin, both of them 12 kDa), lagenin should be classified as a type 1 RIP candidate. α-pisavin and β-pisavin have molecular weights that are also closer to the classical type 1 RIPs than to the small type 1 RIPs, but compared with lagenin, they both have the *N*-glycosidase activity and, in addition, show amino acid similarity with other type 1 RIPs. For that reason, α-pisavin and β-pisavin are considered type 1 RIPs.

Cinphorin is a type 2 RIP from the seeds of *Cinnamomum camphora* with a molecular weight of 46 kDa, which is due to the smaller A-chain than the other classical type 2 RIPs [368]. It is proposed that cinphorin is a cleaving product of cinnamomin, another type 2 RIP from *Cinnamomum camphora*, or its mRNA [367]. Cleaving processes during the evolution of RIPs are not unusual [29], but cinphorin is the only type 2 RIP with a smaller A-chain known to date, and, therefore, it is questionable whether it is necessary to denominate an extra classification for cinphorin. Considering that there might be more RIPs that are not detected to date, of which one could be another type 2 RIP with a smaller A-chain, however, we propose to classify cinphorin as a “small type 2 RIP” (sRIP 2).

**Table 2:** Small RIPs.

Protein	Source	Mw	Classification	References
α-benincasin	*Benincasa hispida* (Cucurbitaceae)	12 kDa	sRIP 1	[151]
β-benincasin	*Benincasa hispida* (Cucurbitaceae)	12 kDa	sRIP 1	[151]
Charantin	*Momordica charantia* (Cucurbitaceae)	9.7 kDa	sRIP 1	[216]
Cinphorin	*Cinnamomum camphora* (Lauraceae)	46 kDa	sRIP 2	[367,368]
Luffacylin	*Luffa cylindrica* (Cucurbitaceae)	7.8 kDa	sRIP 1	[184]
Luffangulin	*Luffa acutangula* (Cucurbitaceae)	5.6 kDa	sRIP 1	[174]
Luffin P1	*Luffa cylindrica* (Cucurbitaceae)	5226.1 Da	sRIP 1	[185]
γ-momorcharin	*Momordica charantia* (Cucurbitaceae)	11.5 kDa	sRIP 1	[205]
S-trichokirin	*Trichosanthes kirilowii* (Cucurbitaceae)	8 kDa	sRIP 1	[259]
Trichokirin S1	*Trichosanthes kirilowii* (Cucurbitaceae)	11426 Da	sRIP 1	[258]
Trichosanthrip	*Trichosanthes kirilowii* (Cucurbitaceae)	10964 Da	sRIP 1	[256]

**Table 3:** RIP candidates and RIP-like proteins.

Protein	Source	Mw	IC_50_	Classification	References
*A. ellenbeckii* lectin	*Adenia ellenbeckii* (Passifloraceae)	60 kDa	10.1 µg/mL; 1.2 µg/mL	RIP 2 candidate	[45]
*A. glauca* lectin	*Adenia glauca* (Passifloraceae)	n.a.	>10 µg/mL; >5 µg/mL	RIP 2 candidate	[45]
*A. keramanthus* lectin	*Adenia keramanthus* (Passifloraceae)	60–65 kDa	10.0 µg/mL; 1.1 µg/mL	RIP 2 candidate	[45]
*A. spinosa* lectin	*Adenia spinosa* (Passifloraceae)	n.a.	4.7 µg/mL; 0.8 µg/mL	RIP 2 candidate	[45]
*A. venenata* lectin	*Adenia venenata* (Passifloraceae)	60 kDa	2.4 µg/mL; 0.4 µg/mL	RIP 2 candidate	[45]
Arborvitae RIP	*Thuja occidentalis* (Cupressaceae)	n.a.	n.a.	RIP candidate	[272]
BDA	*Bryonia cretica* subsp. *dioica* (Cucurbitaceae)	61 kDa	>1500 nm	RIP 2-like lectin	[73,156]
*Boerhaavia* inhibitor	*Boerhaavia diffusa* (Nyctaginaceae)	16–20 kDa	n.a.	RIP 1 candidate	[371,372,373]
CA-SRI	*Clerodendrum aculeatum* (Lamiaceae)	34 kDa	<0.01 nM	RIP 1 candidate	[358,359]
CF-RIP	*Cucumis ficifolius* (Cucurbitaceae)	n.a.	n.a.	RIP 1 candidate	[159]
CIP-34	*Clerodendrum inerme* (Lamiaceae)	34 kDa	87.4 nM; 3 µg/mL	RIP 1 candidate	[360,361]
CIP31	*Nicotiana tabacum* (Solanaceae)	31 kDa	n.a.	RIP 1-like protein	[460]
Crotin I	*Croton tiglium* (Euphorbiaceae)	40 kDa	n.a.	RIP 1 candidate	[273,275]
Cucurmoschin	*Cucurbita maxima* (Cucurbitaceae)	9 kDa	1.2 µM	small RIP 1 candidate	[160]
Foetidissimin	*Cucurbita foetidissima* (Cucurbitaceae)	63 kDa	25.9 nM	peculiar RIP 2	[157]
Lagenin	*Lagenaria siceraria* (Cucurbitaceae)	20 kDa	0.21 nM	RIP 1 candidate	[171]
Leonurin	*Leonurus japonicus* (Laminariaceae)	n.a.	n.a.	RIP candidate	[362]
Luffin-S	*Luffa cylindrica* (Cucurbitaceae)	10 kDa	0.34 nM	small RIP 1 candidate	[186]
LuffinS(1)	*Luffa cylindrica* (Cucurbitaceae)	8 kDa	130 nM	small RIP 1 candidate	[187]
LuffinS(2) = luffin S2	*Luffa cylindrica* (Cucurbitaceae)	7.8 kDa	10 nM	small RIP 1 candidate	[187,188]
LuffinS(3)	*Luffa cylindrica* (Cucurbitaceae)	8 kDa	630 nM	small RIP 1 candidate	[187]
Malanin	*Malania oleifera* (Olacaceae)	61,875 Da	n.a.	lectin/RIP 2 candidate	[384]
ε-momorcharin	*Momordica charantia* (Cucurbitaceae)	24 kDa	170 nM	RIP 1 candidate	[203]
α-moschin	*Cucurbita moschata* (Cucurbitaceae)	12 kDa	17 µM	small RIP 1 candidate	[168]
β-moschin	*Cucurbita moschata* (Cucurbitaceae)	12 kDa	300 nM	small RIP 1 candidate	[168]
Panaxagin	*Panax ginseng* (Araliaceae)	52 kDa	0.28 nM	peculiar RIP 1 candidate/RNase	[110]
*P. macrocarpa* RIP	*Phaleria macrocarpa* (Thymelaceae)	n.a.	n.a.	RIP candidate	[462]
Quinqueginsin	*Panax quinquefolius* (Araliaceae)	53 kDa	0.26 nM	peculiar RIP 1 candidate/RNase	[111]
Sativin	*Pisum sativum* var. *macrocarpon* (Fabaceae)	38 kDa	14 µM	RIP 1 candidate	[352]
SGSL	*Trichosanthes anguina* (Cucurbitaceae)	62 kDa	n.a.	RIP 2-like lectin	[234]
SoRIP2	*Spinacia oleraceae* (Amaranthaceae)	36 kDa	n.a.	RIP 1 candidate	[106,107]
TCSL	*Trichosanthes cucumerina* (Cucurbitaceae)	69 kDa	n.a.	lectin/RIP 2 candidate	[236]
TDSL	*Trichosanthes dioica* (Cucurbitaceae)	55 kDa	n.a.	lectin/RIP 2 candidate	[270]
TKL-1	*Trichosanthes kirilowii* (Cucurbitaceae)	60 kDa	n.a.	lectin/RIP 2 candidate	[260]
TRIP	*Nicotiana tabacum* (Solanaceae)	26 kDa	100 ng/mL	RIP 1 candidate	[461]

### 3.2. RIP Candidates and RIP-Like Proteins

There are four single chain proteins with a bigger molecular weight than the other type 1 RIPs: Jc-SCRIP from *Jatropha curcas* (38 kDa), β-nigritin from *Sambucus nigra* (40 kDa), sativin from *Pisum sativum* (38 kDa), and CIP-34 from *Clerodendrum inerme* (34 kDa). β-nigritin exhibits *N*-glycosidase activity and, therefore, it is classified as a classic type 1 RIP, because there are no further structural peculiarities [54]. Jc-SCRIP differs not only on the basis of the molecular weight from the other type 1 RIPs, but also with regard to its *N*-terminal amino acid sequence, acidic isoelectric point, high temperature stability, and high sugar content giving this protein additional lectin properties [294]. Because of those unique molecular characteristics, it might be classified as peculiar type 1 RIP as well as b-32 and JIP60. But that would make this issue unnecessarily complicated, because Jc-SCRIP does not have such structural differences compared to other type 1 RIPs like as b-32 and JIP60. Therefore, and because of its *N*-glycosidase activity, Jc-SCRIP is classified as a classical type 1 RIP. Compared with that, sativin and CIP-34 cannot be classified as classical type 1 RIPs, because, among other things, the *N*-glycosidase activity was not found, and, therefore, together with other proteins, they are referred to as “RIP candidates” or “RIP-like proteins” (Table 3). Sativin is considered to be a type 1 RIP candidate, because of its amino acid sequence similarity of 48% to α-pisavin and β-pisavin [352], which are classified as type 1 RIPs as mentioned above. CIP-34 is the major protein of a 100 kDa protein complex with an unknown structure [360]. In Girbés *et al.* [7], it is indeed classified as a classical type 1 RIP, but it might be better to assign CIP-34 to the peculiar type 1 RIPs, because it is larger than other type 1 RIPs and it consists of protein domains with an unknown structure and function. To be grouped into the RIPs, however, the *N*-glycosidase activity of CIP-34 has to be detected. Thus, it is classified as type 1 RIP candidate until further notice.

Panaxagin from *Panax ginseng* and quinqueginsin from *Panax quinquefolius* are two other proteins that differ from the classical type 1 RIPs with regard to molecular weight, structure, and functionality. Both panaxagin and quinqueginsin are homodimeric proteins with molecular weights of 52 kDa and 53 kDa, respectively [110,111]. The amino acid sequence of panaxagin and quinqueginsin show similarities with both RNases and type 1 RIPs, and on the basis of their high translation-inhibitory activities of 0.26 nM and 0.28 nM, respectively, they are classified as RIPs, where the authors proposed the denomination “dimeric type 1 RIP”. Due to their unusual dimeric structure, they can also be considered as peculiar type 1 RIPs. As mentioned above, the *N*-glycosidase activity of a protein needs to be detected in order to be classified as an RIP, but this was not possible for either panaxagin or quinqueginsin, because they both show strong RNase activity destroying the ribosomes. Therefore, both panaxagin and quinqueginsin are considered as peculiar type 1 RIP candidates until the whole amino acid sequence is analyzed, which will or will not show the conserved amino acids of the active site region.

SoRIP2 from *Spinacia oleraceae* is a type 1 RIP candidate, because the *N*-glycosidase activity assay was not performed, but the amino acid sequence shows similarities to the shiga/ricin toxic domain [106]. Interestingly, SoRIP2 only shows low sequence similarity with SoRIP1, another protein from *Spinacia oleraceae* that is classified as type 1 RIP.

*Boerhaavia* inhibitor from *Boerhaavia diffusa* was described as a virus inhibitor without mentioning any more details about the inhibitory activity of rabbit reticulocyte lysate or *N*-glycosidase activity [371,372]. But the size of 16–20 kDa and the fact that antiserum against the type 1 RIP MAP from *Mirabilis jalapa* giving positive reaction with *Boerhaavia diffusa* extract [373], led us to the conclusion to denote *Boerhaavia* inhibitor as a RIP 1 candidate.

CA-SRI from *Clerodendrum aculeatum* is like *Boerhaavia* inhibitor an antiviral protein that induces systemic resistance [358]. Neither the inhibition of translation of rabbit reticulocyte lysate nor the *N*-glycosidase was demonstrated, but the size of 34 kDa and the amino acid sequence homology of 54% [359] to the type 1 RIP PAP from *Phytolacca americana* make CA-SRI a RIP 1 candidate.

CF-RIP is a type 1 RIP candidate from *Cucumis ficifolius* that was obtained by cloning and sequencing the cDNA [159]. To be classified as type 1 RIP, native CF-RIP has to be isolated as well as the *N*-glycosidase activity has to be detected. Compared with that, the enzymatic activity of ε-momorcharin from *Momordica charantia* indeed was detected, but it was not denominated as a classical type 1 RIP, because its IC_50_ of 170 nM is too low. Thus, the authors supposed significant structural dissimilarities of ε-momorcharin from the classical type 1 RIPs [203]. Another protein showing *N*-glycosidase activity, but is not classified as type 1 RIP, is TRIP from *Nicotiana tabacum*, because TRIP releases less adenine compared to type 1 RIPs [461]. It shows almost all the characteristics of type 1 RIPs instead of sequence similarity with other type 1 RIPs, wherein it should be mentioned that only 15 internal amino acids were analyzed. The authors classified TRIP as a RIP-like protein, but the fact that it shows superoxide dismutase activity, that is well known for RIPs [23], led us to the proposal to classify TRIP as a type 1 RIP candidate until the whole amino acid sequence is analyzed, which will or will not show the conserved amino acids of type 1 RIPs. Another protein from *Nicotiana tabacum* is CIP31 that shows a distinct mechanism of action as RIPs. In addition, not only is its *N*-terminal amino acid sequence different from the RIPs, but it is also only expressed with the presence of Cinchonaglykoside C (1) [460]. Thus, it is denominated as an RIP-like protein.

Because of cleaving supercoiled DNA by a crude extract of seeds from *Phaleria macrocarpa*, it was assumed that at least one RIP is included in this extract [462], but there were no more details given about this assumed RIP. The same applies to arborvitae RIP, where it is only known that there is probably a RIP synthesized by arborvitae [272], but we could only find the abstract of this paper during our investigation and in the abstract it is not clarified whether it is a RIP or just an RNase. Due to a lack of any further details, we propose to denominate these assumed RIPs as RIP candidates without mentioning the more detailed denomination RIP 1 or RIP 2 candidate. The same applies to leonurin from *Leonurus japonicus*, for which we did not find any further information as well [362].

As mentioned in the introduction, some lectins were found from several *Adenia* species [45], of which the lectins from *Adenia lanceolata* and from *Adenia stenodactyla* were classified later as type 2 RIPs and were denominated as lanceolin and stenodactylin [391], respectively. The lectin from *Adenia goetzei* is a type 2 RIP as well, because it was found that it is active as glycosylase, which releases adenine from herring sperm DNA [464]. On the other hand, the lectins from *Adenia ellenbeckii*, *Adenia glauca*, *Adenia keramanthus*, *Adenia spinosa*, and *Adenia venenata* indeed consist of two protein chains and inhibit translation in a cell free system, but the *N*-glycosidase activity was not analyzed. Thus, they should be considered as type 2 RIP candidates.

BDA from *Bryonia cretica* subsp. *Dioica*, malanin from *Malania oleifera*, TCSL from *Trichosanthes cucumerina*, TDSL from *Trichosanthes dioica*, and TKL-1 from *Trichosanthes kirilowii* are also two-chain lectins that cannot be clearly classified as type 2 RIPs. All of them have the typical molecular weight of type 2 RIPs and consist of two protein chains resembling the structure of type 2 RIPs that was even shown by X-ray crystallography [260], but the *N*-glycosidase activity assay was not performed excluding BDA. BDA, however, was not inhibitory in the highest tested concentration (IC_50_ > 1500 nM; [73]). These samples show that proteins having both a similar molecular weight and molecular structure, but lacking *N*-glycosidase activity, cannot be classified as classical type 2 RIPs. Therefore, we propose to classify BDA as a type 2 RIP-like protein and malanin, TCSL, and TDSL as type 2 RIP candidates, because the *N*-glycosidase activity of these proteins could neither be confirmed nor excluded to date.

At this point two other proteins should be mentioned differing from the classical type 2 RIPs or two-chain lectins with regard to the molecular structure: Foetidissimin from *Cucurbita foetidissima* and SGSL from *Trichosanthes anguina*. Foetidissimin indeed inhibits translation by acting as *N*-glycosidase and it consists of two protein chains, but these chains are not held together through a disulphide bridge [157]. This is hitherto unique for type 2 RIPs and, therefore, we propose to classify foetidissimin as a peculiar type 2 RIP on the basis of the denomination for the peculiar type 1 RIP b-32. The A-chain of SGSL is cleaved obtaining two non-covalently linked components A_α_ and A_β_-s-s-B. Thus, the nucleotide and carbohydrate-binding sites of SGSL are changed and compared to cinphorin, SGSL does not show *N*-glycosidase activity, which is due to the cleaved A-chain, but, as X-ray crystallography shows a very similar molecular structure compared to type 2 RIPs, SGSL is classified as a type 2 RIP-like protein. As mentioned above, cleaving processes are not unusual for RIPs, so it was shown that TrSNA-I and TrSNA-If, both lectins from *Sambucus nigra*, are cleaving products of the type 2 RIPs SNA-I and SNA-If, respectively. This supports the hypothesis that certain lectins and type 2 RIPs must be evolutionarily related.

### 3.3. Dimeric, Tetrameric, and Octameric Type 2 RIPs and Dimeric Lectins

Most of the dimeric, tetrameric, and octameric type 2 RIPs or dimeric lectins are synthesized by plant species belonging to the *Sambucus* genus, which are reviewed in Ferreras *et al.* [29] and Ferreras *et al.* [72]. In these reviews, the proteins are grouped in “heterodimeric type 2 RIPs”, “tetrameric type 2 RIPs”, “monomeric lectins”, and “homodimeric lectins”. The heterodimeric type 2 RIPs represent the classical type 2 RIPs consisting of one A-chain and one B-chain linked together through a disulphide bridge [A-s-s-B]. Tetrameric type 2 RIPs consist of four protein chains and, therefore, the proposal was made to denominate these proteins as type 4 RIPs [306]. But that would mean that there are type 1, type 2, and type 4 RIPs, but no type 3 RIPs, because they were renamed peculiar type 1 RIPs, which may lead to confusion. Thus, we agree with the term “tetrameric type 2 RIPs”. These RIPs are subdivided into two groups. One of those consist of two [A-s-s-B]-units linked together non-covalently, which can also be considered dimeric classical type 2 RIPs ([A-s-s-B]_2_). It should be mentioned that the [A-s-s-B]-units can be different, e.g., in RCA from *Ricinus communis* ([A-s-s-B]_α_[A-s-s-B]_β_; [316,323]). The other group of tetrameric type 2 RIPs includes proteins with an extra disulphide bond between the two B-chains [A-s-s-B-s-s-B-s-s-A]. In Ferreras *et al.* [72], SNA-I and SNA-If were grouped herein, but it was shown that both native SNA-I and native SNA-If occur as a 240 kDa protein having the structure [A-s-s-B-s-s-B-s-s-A]_2_ [69]. Thus, these proteins can also be considered as dimeric tetrameric type 2 RIPs linked non-covalently, but we propose the denomination octameric type 2 RIPs. PMRIPt from *Polygonatum multiflorum* and abrin from *Abrus precatorius* are also octameric type 2 RIPs consisting of four [A-s-s-B]-units, which are linked non-covalently as well ([A-s-s-B]_4_; [117,328]). They can also be considered as tetrameric classical type 2 RIPs.

Dimerization or oligomerization is a common behavior of purified and concentrated proteins. To avoid any confusion, the denomination of tetrameric type 2 RIPs with the structure [A-s-s-B]_2_ and octameric type 2 RIPs is not meant as a real classification, because this would separate closely related type 2 proteins such as SNAI and SSA or abrin and pulchellin. We grouped those proteins in Table 4 as an addition to Table 1 to explain the bigger molecular weights and to show their native form in which they have been detected.

**Table 4:** Dimeric, tetrameric, and octameric type 2 RIPs and dimeric lectins.

Structure	Protein	Source	Mw	References
Octameric **[A-s-s-B-s-s-B-s-s-A]_2_**	SNA-I	*Sambucus nigra* (Adoxaceae)	240 kDa	[66,69]
	SNA-If	*Sambucus nigra* (Adoxaceae)	240 kDa	[69]
Octameric **[A-s-s-B]_4_**	Abrin	*Abrus precatorius* (Fabaceae)	260 kDa	[328]
	PMRIPt	*Polygonatum multiflorum* (Asparagaceae)	240 kDa	[117]
Tetrameric **[A-s-s-B-s-s-B-s-s-A]**	SEA	*Sambucus ebulus* (Adoxaceae)	135,630 Da	[50]
	SNAflu-I	*Sambucus nigra* (Adoxaceae)	subunits of 30–33 kDa	[71,72]
	SRA	*Sambucus sieboldiana* (Adoxaceae)	120 kDa	[79]
	SSA	*Sambucus sieboldiana* (Adoxaceae)	160 kDa	[81]
Tetrameric **[A-s-s-B]_2_**	APA	*Abrus precatorius* (Fabaceae)	126–134 kDa	[315,341,342,345]
	*Hura crepitans* latex lectin	*Hura crepitans* (Euphorbiaceae)	112 kDa	[279]
	MCL	*Momordica charantia* (Cucurbitaceae)	115–124 kDa	[207,218,219,220]
	ML-I	*Viscum album* (Santalaceae)	115–125 kDa	[445,447,450,451,452]
	Nigrin b	*Sambucus nigra* (Adoxaceae)	120 kDa	[58]
	Nigrin f	*Sambucus nigra* (Adoxaceae)	120 kDa	[62]
	SNA-I’	*Sambucus nigra* (Adoxaceae)	120 kDa	[67]
Tetrameric **[A-s-s-B]_α_[A-s-s-B]_β_**	RCA	*Ricinus communis* (Euphorbiaceae)	118–130 kDa	[316,323]
Homodimeric lectins **[B]_2_**	*E. characias* lectin	*Euphorbia characias* (Euphorbiaceae)	80 kDa	[279]
	*Luffa acutangula* fruit lectin	*Luffa acutangula* (Cucurbitaceae)	48 kDa	[175]
	Protein fraction 1	*Momordica charantia* (Cucurbitaceae)	49 kDa	[224]
	Protein fraction 2	*Momordica charantia* (Cucurbitaceae)	49 kDa	[224]
	*Sechium edule* fruit lectin	*Sechium edule* (Cucurbitaceae)	44 kDa	[230]
	SELld	*Sambucus ebulus* (Adoxaceae)	67,906 Da	[52]
	SELfd	*Sambucus ebulus* (Adoxaceae)	68 kDa	[47]
	SNAld	*Sambucus nigra* (Adoxaceae)	n.a.	[63]

### 3.4. Non-Toxic Type 2 RIPs

For a long time, all type 2 RIPs were considered to be highly potent toxins, but, to date, there are also known type 2 RIPs, which are not or only less toxic *in vivo*, and therefore they are denominated as non-toxic type 2 RIPs (reviewed in [7,8], not listed in this review). Nearly all of them have lectin properties and show *N*-glycosidase activity in a cell-free system, so that these characteristics cannot be the reason for the missing *in vivo*-toxicity. SNLRP1 from *Sambucus nigra* for instance is a non-toxic type 2 RIP without lectin properties. On the other hand, nigrin b from *Sambucus nigra* has lectin properties but is non-toxic as well, because it is degraded rapidly and excreted by cells [8]. Articulatin D from *Viscum articulatum* is another type 2 RIP without lectin properties, but compared to SNLRP1, articulatin D is very toxic [455]. Thus, these examples show that the reasons for the vast differences in toxicity are not clearly understood. Nevertheless, non-toxic type 2 RIPs are quite interesting for anti-cancer therapy, because they may have a lower potential of side effects.

### 3.5. Demotion of Some RIPs

At last, it should be mentioned that there are some proteins, which were first classified as RIPs, but it was later shown that they act with a different mechanism of action for inhibiting translation than *N*-glycosidase. Melonin from *Cucumis melo* was first classified as type 1 RIP [465], but a few years later, it was found that it is a ribonuclease (RNase) that specifically degrades poly(C)- and cytidine-containing bonds [466]. Crotin I and crotin II, two proteins from *Croton tiglium*, were classified as type 1 RIPs as well [7], but for crotin II, it was found that it belongs to RNA hydrolases, which cleave a phosphodiester bond between G^4325^ and A^4326^ of 28S rRNA [10]. That is why crotin II is not listed in Table 1. Crotin I is a 40 kDa protein that does not fit into the type 1 RIP classification with regard to the molecular weight and in addition, its *N*-glycosidase activity was also not detected, because the corresponding assay was not performed [273,274]. Thus, the *N*-glycosidase activity cannot be excluded and, therefore, crotin I should be classified as a type 1 RIP candidate. At this point, it should be mentioned that there is a type 1 RIP with *N*-glycosidase activity against bacterial rRNA [277], which was denominated as crotin 2. The denomination of crotin II and crotin 2 may lead to confusion particularly in Girbés *et al.* [7], as crotin I and crotin II are also denominated as crotin 2 and crotin 3, respectively. For that reference, however, we could not find any proof and, therefore, in Table 1, we listed crotin I and crotin 2 separately, but we did not list crotin II on the basis of the reasons mentioned above and also excluded crotin 3, because too little information exists. The question remains as to whether there are more RIPs which should be demoted.

## 4. Conclusions

Hitherto, several approaches concerning the nomenclature of RIPs were proposed. Most of the proteins were denominated by using a part of the genus or species name followed with the ending “-in”, e.g., agrostin from *Agrostemma githago* or ocymoidin from *Saponaria ocymoides*. If there is more than one RIP synthesized by the same plant, the denominations are followed by an Arabic or Roman numeral, e.g., asparin 1 and asparin 2 from *Asparagus officinalis* or pulchellin PI, pulchellin PII, and pulchellin PIII from *Abrus pulchellus*. The numerals, however, can also represent the peak number, in which the proteins were eluted, e.g., agrostin 2, agrostin 5, and agrostin 6 [112]. Some proteins are denominated with additional information about their molecular weight, e.g., dianthin29 from *Dianthus barbatus* with a size of 29 kDa, or the tissue they are obtained from, e.g., nigrin b from the bark of *Sambucus nigra*. There are also many proteins, which are denominated with abbreviations, mostly using the initials of the genus and species name, e.g., SEA (= *Sambucus ebulus* agglutinin) from *Sambucus ebulus*. At last, modeccin 4B and modeccin 6B from *Adenia digitata* were denominated by using the material for their isolation. Modeccin 4B was isolated by affinity chromatography on Sepharose 4B and modeccin 6B was isolated by affinity chromatography on acid-treated Sepharose 6B [390].

In 1996, an unambiguous nomenclature was already demanded [58], but today there is still not a uniform classification existing for RIPs. This may be due to the fact that there are several exceptions of RIPs and RIP related proteins, which cannot be grouped into the classical type 1 or type 2 RIPs concerning the structure and/or function of these proteins. Besides the small RIPs, which were already designated in 1996 [205], we propose the term “RIP candidate” for those proteins, which are structurally related to the classical type 1 and type 2 RIPs and/or inhibit translation, but were not analyzed with regard to their *N*-glyosidase activity. On the other hand, ε-momorcharin is also a RIP candidate [203], which is indeed active as *N*-glycosidase but shows significant structural dissimilarities from the classical RIPs. These “RIP candidates” can be subdivided into small type 1 RIP (e.g., cucurmoschin), type 1 RIP (e.g., sativin) or type 2 RIP candidates (e.g., malanin) concerning the molecular weight and structure.

For the denomination of those proteins which cannot be grouped into the classic small RIPs, type 1 RIPs or type 2 RIPs due to their unusual structure, but act as *N*-glycosidase (b-32 and JIP60), we agree with the term “peculiar RIP” [7,8], and, therefore, we add the peculiar type 2 RIP foetidissimin, which lacks the disulphide bridge between the A-chain and B-chain. Because of the dimeric structure of panaxagin and quinqueginsin, they should be considered as peculiar type 1 RIPs, or, more precisely, as peculiar type 1 RIP candidates, because the *N*-glycosidase activity could not be analyzed, but they show amino acid sequence similarities with other type 1 RIPs.

All other proteins, which are structurally related to RIPs but lack *N*-glycosidase activity, should be referred to as RIP 1-like or RIP 2-like proteins/lectins.
